# Harvest time affects antioxidant capacity, total polyphenol and flavonoid content of Polish St John’s wort’s (*Hypericum perforatum* L.) flowers

**DOI:** 10.1038/s41598-021-83409-4

**Published:** 2021-02-17

**Authors:** Katerina Makarova, Joanna J. Sajkowska-Kozielewicz, Katarzyna Zawada, Ewa Olchowik-Grabarek, Michał Aleksander Ciach, Krzysztof Gogolewski, Natalia Dobros, Paulina Ciechowicz, Hélène Freichels, Anna Gambin

**Affiliations:** 1 Department of Physical Chemistry, Chair of Physical Pharmacy and Bioanalysis, Faculty of Pharmacy With Laboratory Medicine Division, Medical University of Warsaw, Banacha 1, 02-097 Warsaw, Poland; 2 Laboratory of Molecular Biophysics, Department of Microbiology and Biotechnology, Faculty of Biology, University of Bialystok, Ciolkowskiego 1J, 15-245 Bialystok, Poland; 3 Faculty of Mathematics, Informatics and Mechanics, University of Warsaw, Stefana Banacha 2, 02-097 Warszawa, Poland; 4 Centre for Statistics, Hasselt University, Diepenbeek, 3590 Limburg, Belgium; 5 Magritek GmbH, Philipsstraße 8, 52068 Aachen, Germany

**Keywords:** Mathematics and computing, NMR spectroscopy, Natural variation in plants

## Abstract

The polyphenol content and antioxidant capacity of hyperforin and hypericin-standardized *H. perforatum* L. extracts may vary due to the harvest time. In this work, ethanol and ethanol–water extracts of air-dried and lyophilized flowers of *H. perforatum* L., collected throughout a vegetation season in central Poland, were studied. Air-dried flowers extracts had higher polyphenol (371 mg GAE/g) and flavonoid (160 mg CAE/g) content, DPPH radical scavenging (1672 mg DPPH/g), ORAC (5214 µmol TE/g) and FRAP (2.54 mmol Fe^2+^/g) than lyophilized flowers extracts (238 mg GAE/g, 107 mg CAE/g, 1287 mg DPPH/g, 3313 µmol TE/g and 0.31 mmol Fe^2+^/g, respectively). Principal component analysis showed that the collection date influenced the flavonoid and polyphenol contents and FRAP of ethanol extracts, and DPPH and ORAC values of ethanol–water extracts. The ethanol extracts with the highest polyphenol and flavonoid content protected human erythrocytes against bisphenol A-induced damage. Both high field and benchtop NMR spectra of selected extracts, revealed differences in composition caused by extraction solvent and raw material collection date. Moreover, we have shown that benchtop NMR can be used to detect the compositional variation of extracts if the assignment of signals is done previously.

## Introduction

Due to its wide range of well documented pharmacological activities, such as antidepressant, antiviral, and antibacterial effects, St. John’s wort (*Hypericum perforatum* L.) is one of the most consumed medicinal plants in the world^1^. Its extracts are used as phytopharmaceuticals and nutraceuticals.

St. John’s wort antidepressant activity has been related to the synergetic effect of hypericin and phenolic compounds^2^. The latter modulate the key cellular processes such as redox, metabolic and energetic homeostasis, proteostasis, signaling and oxidative stress, thus decreasing the risk of cardiovascular, neurodegenerative and metabolic diseases, as well as of some forms of cancers^3^.

Although, due to the presence of phenolic compounds, *H. perforatum* L. has antioxidant properties, there are only a few studies on this subject^2,4–10^. The variation in total polyphenol (TP) content and antioxidant properties of St. John’s wort from the Balcan peninsula^5^, Lithuania^11,12^, Turkey^13^ and China^14^ was studied. The main factors which influenced the TP and antioxidant properties were geographical origin, whether the plant was wild or cultivated^15^, individual chemotype^16^, part of the plant studied (leaves, flowers, fruits, roots), harvesting stage (floral budding stage, blooming stage or fruit set stage) and the age of the plant (1-, 2- or 3- year plant)^14,17–19^. However, many other factors. including the temperature and light intensity^18,20^ also influence these properties.

Still, there is a lack of studies on compositional variation within one season for wild plants from Poland, which is the 3rd largest European market of *H. perforatum* L. products. There is almost no data concerning the potential influence of drying method, i.e. air-drying and lyophilization (freeze-drying), despite the application of both in *Hypericum* studies. To the best of our knowledge, only one such study is available, comparing the hot-air and freeze drying approach in relation to antioxidant properties^21^.

TP and antioxidant properties are very general characteristics of plant extracts, typically studied by chromatographic techniques. These techniques require reference compounds and usually do not reveal unknown metabolites that may contribute to the biological activity of the phytochemicals^22^. Nuclear Magnetic Resonance (NMR) spectroscopy allows to overcome these limits, detecting both known and unknown constituents of complex mixtures. In particular, 1H NMR spectroscopy is widely used in studies of plant extracts to quantitatively and simultaneously analyze all proton-bearing compounds, and consequently all relevant substance classes in the extracts^22,23^.

The signals of main components of H. perforatum L. extracts were assigned previously using high field NMR spectra^24^.

Handling a high field NMR spectrometer is sophisticated and costly, and requires large quantities of non-environmentally friendly liquid helium and liquid nitrogen. Thus, in the last decades, benchtop instruments are gaining popularity. A number of benchtop NMR spectrometers operating at 40-100 MHz is available and used both in research and industry. Benchtop NMR spectrometers are significantly cheaper, smaller and much easier in operation, and do not require liquid helium nor liquid nitrogen. However, due to their low resolution, benchtop spectrometers are used mainly in chemical reaction monitoring, studies of synthetic drugs, and other applications where well separated signals can be obtained. On the other hand, typical NMR spectra of plant extracts consist of highly overlapping signals. Low operating frequencies increase the overlap even further, resulting in signals which are hard to identify or quantify. That limits the direct application of benchtop NMR for the studies of plant extracts.

The combination of high field NMR with chemometric methods is commonly applied in the field of metabolomics profiling, food technology^25^ and various plant extract studies^26^. However, there are no studies showing a combination of benchtop NMR with chemometric methods for the studies of plant extracts.

This work aims to study the seasonal variation of TP and TF content and antioxidant properties in various *H. perforatum* L. extracts obtained from both air-dried and lyophilized flowering tops. The antioxidant properties were studied with DPPH, ORAC and FRAP tests, which in combination with chemometric methods, are widely used to screen medicinal herbs^27^.

It was reported that, due to their antioxidant properties, polyphenol-rich extracts may protect erythrocytes from oxidative damage^28,29^. However, in order to verify such hypothesis, standard tests performed in vitro need to be followed by studies in biological models^30^. Using an erythrocyte-based model of BPA-induced oxidative stress, we have verified the antioxidant properties of samples that exhibited the highest antioxidant properties in DPPH, ORAC and FRAP tests.

We have tested the use of benchtop NMR for compositional variation studies. We have applied statistical methods to check if a benchtop instrument accurately reflects the differences in samples as observed in high-field spectra.

Our proposed approach to tracking changes in the composition of plant extracts, based on a combination of benchtop NMR, statistical, and chemometric methods, could extend the range of applications of benchtop NMR from single/pure compounds to the analysis of complex mixtures. It is applicable to other composition variation studies of other plant extracts.

The information about variation of TP and TF, as well as of antioxidant capacity of *H. perforatum* L. from Poland, is complementary with other studies of *H. perforatum* L. This is important for new applications of St. John’s Wort extracts as a functional food ingredient.

## Results and discussion

The average extraction yield for ethanol extracts was 16% for air-dried plant material and 17% for lyophilized plant material, while for ethanol–water extracts it was 28% for air-dried plant material and 19% for lyophilized plant material. It was not influenced by the water content of dried plant material, which was 37.3 ± 1.7% f or air-dried and 4.3 ± 0.2% for lyophilized flowers.

### Polyphenol and flavonoid content

**Figure 1:**
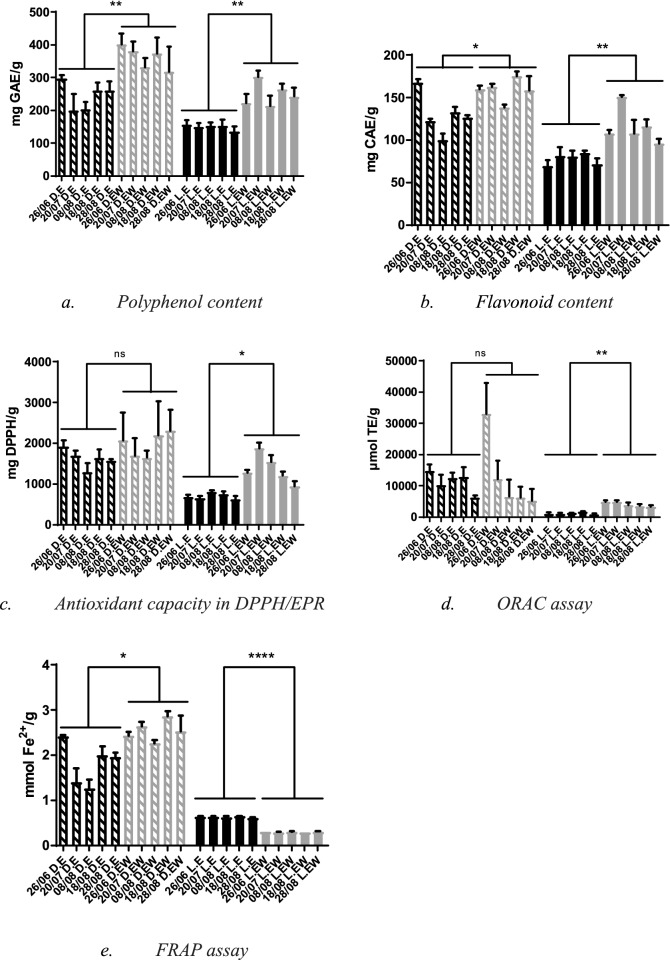
Polyphenol **(a)** and flavonoid content **(b)** and antioxidant capacity in DPPH/EPR test **(c)**, in ORAC assay **(d)** and in FRAP assay **(e)** in ethanol (E, black) and ethanol–water (EW, gray) extracts prepared from air-dried (D, stripes) and lyophilized (L, filled) flowers of *H. perforatum* L. for different harvest time (26.06, 20.07, 08.08, 18.08 and 28.08). The values are means with standard deviation). In the figure mean values with standard deviation are denoted. Additionally, to encode the statistical significance asterisks are used: (****)—p < 0.0001; (***)—p < 0.001; (**)—p < 0.01; (*)—p < 0.05; (ns)—p 0.05.

Ethanol–water extracts prepared from the air-dried material had the highest TP content (median 371 ± 49 mg GAE/g, range from 317.6 to 402.2 mg GAE/g), followed by the ethanol extracts (median 245 ± 26 mg GAE/g, range from 199.6 to 298.2 mg GAE/g) also prepared from the air-dried material (Fig. 1a, Table S1). Moreover, these ethanol and ethanol–water extracts showed a clear dependency of TF and TP content on the harvest time.

The statistically significant maxima of TP content for ethanol extracts prepared from air-dried materials were at 26.06 (Table S2), whereas the statistically significant maxima of TP content for ethanol–water extract were at 26.06 and 18.06 (Table S2).

Extracts prepared from the lyophilized plant material had lower TP content than those of extracts prepared from air-dried material. Still, the ethanol–water extracts had significantly higher TP content (median 238 ± 26 mg GAE/g, range from 214.2 to 302.4 mg GAE/g) than the ethanol extracts (median 152 ± 13 mg GAE/g, range from 135.7 to 156.1 mg GAE/g). The harvest time affected TP content of ethanol–water extracts only (Fig. 1a, Table S1). The TP content in ethanol–water extract at 20.07 is significantly higher than at all other collection dates (Table S2), whereas ethanol extracts from all collection dates have similar TP content (Table S2) except for 28.08, where it is significantly lower. Previous studies reported 142 mg GAE/g and 222 mg GAE/g of the TP content of H. perforatum L. ethanol–water extracts from Romania^31^ and Bulgaria^30^, respectively. The TP content for the extracts prepared from lyophilized flowers reported herein is within the expected ranges, whereas the TP content for the extracts prepared from dried flowers is higher than reported previously.

Such variation of total polyphenols in extracts prepared from dried material could be explained by higher concentrations of polyphenols and flavonoids in flowers than in other aerial parts of the plant which were used in previously reported studies. Umek et al. showed a significant difference in the concentration of rutin, hyperoside, hypericin, hyperforine and other bioactive compounds between leaves and flowers of *H. perforatum* L. from Slovenia^32^. Also, Cirak et al. reported differences between hypericin, chlorogenic acid and the flavonoids content in different parts and development stages of the plant^33^. It was shown that the maximum total polyphenol content is observed in the herb (leaves and stems) at the floral-budding and full flowering stage^13^. In this work, we have used plant material at the flowering stage of development, which may have contributed to the high TP content observed.

The TF content of ethanol and ethanol–water extracts prepared from air-dried samples (122 ± 4 mg CAE/g and 160 ± 7 mg CAE/g, with ranges 100.4–167.8 mg CAE/g for ethanol and 138.4–175.3 mg CAE/g for ethanol–water extracts) were higher than for those prepared from lyophilized material (median 80 ± 7, range 69.6–85.0 mg CAE/g and median 107 ± 16 mg CAE/g, range 96.0–151.0 mg CAE/g, respectively) (Fig. 1b, Table S1). In both groups, the differences in TF content between ethanol and ethanol–water extracts were statistically significant.

The main flavonoids in the ethanol extracts are reported to be rutin, hyperoside quercitrin, quercetin and apigenin^11,30^. However, their content could vary up to 100 times^11,34^.

Total flavonoid content of ethanol–water extracts of aerial parts of *H. perforatum* L. collected in Bulgaria reported by Katsarova et al. (62.36 ± 0.71 mg CAE/g) is lower than total flavonoid content reported here for the extracts prepared from dried and lyophilized flowers^30^.

Previously, it was shown that air-drying at the room temperature in a dark place did not change the flavonoid content as compared with freshly collected plant material, except for the physiologically younger plant material (first year of cultivation, first cut) which showed significant increase in flavonoid content after air-drying^15,35^.

It is believed that lyophilization (freeze-drying) of plant material preserves more large-molecular-weight condensed tannins than air-drying^36^. However, it was reported that in some cases freeze-drying could decrease polyphenol content^36^. For example, it was shown that *Cosmos caudatus* extracts prepared from air-dried plant material had a higher polyphenol content and antioxidant activity than extracts prepared from frozen and lyophilized plant material^37^. Similarly, in this work, we have observed that *H. perforatum* L. extracts prepared from the lyophilized flowers had lower polyphenol and flavonoid content.

The harvest time dependencies of TF content were observed in all samples. The statistically significant maxima of TF content for ethanol and ethanol–water extracts prepared from air-dried materials were at 28.06 and 18.08, respectively (Table S2). Moreover, the maximum TF in ethanol extracts corresponds to the maximum of TP.

In the ethanol–water extracts prepared from lyophilized material the statistically significant maximum was at 20.07 (Table S2), similarly to the maximum of TP content. On the other hand, for the ethanol extracts from lyophilized material the maximum of TF content was at 18.08 (Table S2).

Seasonal variation in the TP and TP contents could be due to the temperature and water stress. It was shown that high temperature (35 ºC) leads to no flowering, whereas in the temperature range from 20 to 30 ºC the hypericin content of flowers changed from 0.6 to 1.2 mg/g fresh weight^38^. Also, it was shown that under water stress major biochemical and physiological changes took place, e.g. an increase in hyperforin concentration and a decrease in hypericin content. Moreover, it was shown that changes in the biochemical profile of the plant did not recover after irrigation^39^. Based on weather information available for Central Poland in 2016 (Table S3), the variations of the average air temperatures were significant for *H. perforatum* L. (from 22 to 29 ºC) and could potentially affect the TP and TF content.

### Antioxidant capacity

#### DPPH

Among the extracts prepared from the dried flowers, the difference in DPPH radical scavenging of the ethanol–water extracts (median 1672 mg DPPH/g, range 1647–2306 mg DPPH/g) and the one of the ethanolic extracts (median 1376 mg DPPH/g, range 1290—1920 mg DPPH/g) (Fig. 1c, Table S4) was not statistically significant. Extracts prepared from the lyophilized flowers showed lower DPPH radical scavenging activity. However, the ethanol–water extracts had significantly higher DPPH radical scavenging activity than those of ethanol extracts (median 1287 mg DPPH/g and 653 mg DPPH/g, respectively) (Fig. 1c, Table S4). Our results are close to those reported by^19^ (recalculated value 1201 mg DPPH/g).

In the extracts prepared from lyophilized flowers, the time of collection affected mainly DPPH radical scavenging activity of the ethanol–water extracts. The highest DPPH radical scavenging activity was observed for the extracts prepared from flowers collected on 20.07 and 8.08. (Table S5). For ethanol extracts, the highest DPPH radical scavenging activity was observed for the lyophilized material collected on 08.08 (Table S5).

Previously reported DPPH radical scavenging activity for ethanol or ethanol–water extracts of *H. perforatum* L. ranged from 9.85 mg DPPH/g^31^ to 10,500 mg DPPH/g^9^. Such a large variation in the DPPH radical scavenging values could be due to different plant parts used for the extract preparation and different extraction methods.

#### ORAC

The ORAC median values of ethanol (9701 µmol TE/g) and ethanol–water extracts (5214 µmol TE/g) of *H. perforatum* L. prepared from the dried flowers are rather similar (Table S4). The range of ORAC values for ethanol extracts was 6325–14,713 μmol TE/g while the range for ethanol–water extracts was 5141–33,111 μmol TE/g. The total antioxidant capacity of the ethanol–water extracts prepared from flowers, observed at the end of June (33,111 µmol TE/g), was statistically significantly higher than for all other collection dates (Fig. 1d, Table S5). Among the extracts prepared from the lyophilized flowers, the ORAC median values obtained for the ethanol–water extracts (3313 µmol TE/g) were higher than those observed for ethanol extracts (775 µmol TE/g) (Table S4). The range of ORAC values for ethanol extracts of lyophilized flowers was 863–1704 μmol TE/g while the range for ethanol–water extracts was 3282–4893 μmol TE/g.

Previously, the total antioxidant capacity of the *H. perforatum* L. ethanolic extracts was reported to be 5950.5 ± 328.4 µmol TE/g, which was mainly due to the presence of rutin, hyperoside, quercetin and apigenin^30^. As the total amount of these four flavonoids was reported to be lower (62.36 ± 0.71 mg/g) than we observed for TF in this work, we could expect larger ORAC values.

#### FRAP

The FRAP values of the extracts prepared from the dried material were higher than those of the extracts prepared from the lyophilized flowers (Fig. 1e) (from 1.26 ± 0.20 to 2.42 ± 0.20 mmol Fe^2+^/g for ethanol extracts and from 2.27 ± 0.07 to 2.87 ± 0.11 mmol Fe^2+^/g for ethanol–water extracts of air-dried flowers compared with the range of 0.61 ± 0.03 to 0.65 ± 0.01 mmol Fe^2+^/g for ethanol extracts and 0.30 ± 0.01 to 0.31 ± 0.01 mmol Fe^2+^/g for ethanol–water extracts of lyophilized flowers). Among the extracts prepared from the dried flowers, the slightly higher FRAP values were observed for the ethanol–water extract (median 2.54 mmol Fe^2+^/g. Table S4) with the maximum for the extract obtained from flowers collected at 18.08 (Table S5). The maximum FRAP value (2.41 mmol Fe^2+^/g) of ethanol extracts was observed for the extract obtained from flowers collected on 26.06 (Table S5). The FRAP values obtained for the ethanol water extracts were lower than those reported by^19^ (3.7 ± 0.1 mmol Fe^2+^/g). Such high values could be due to the longer time of maceration (72 h) than we used in this work.

In contrast to the air-dried samples, for the extracts prepared from the lyophilized material, the ethanol extracts (median 0.63 mmol Fe^2+^/g) had higher FRAP values than those of ethanol–water extracts (median 0.31 mmol Fe^2+^/g)(Table S4). Our FRAP values for the extracts from the lyophilized flowers are close to those reported by Stef et al.^31^ (0.21 mmol TE/g, which corresponds to approximately 0.40 mmol Fe2^+^/g).

### Chemometric analysis

Some studies report good correlation of TP content with the antioxidant capacity of the extracts from various plants. It is also true for the *H. perforatum* L. extract when it is analyzed in one set with other plant extracts. Katsarova et al.^30^ reports correlation of polyphenol content with ORAC values for 6 medicinal plants including *H. perforatum* L. Other authors reported good correlation of TP with FRAP values for 10 and 70 plant extracts and weak correlation of TP with DPPH radical scavenging^40,41^.

However, for the *H. perforatum* L. extracts, such correlation is not clear. Božin et al.^5^ reported a good correlation of DPPH radical scavenging with the content of TP and TF for the *Hypericum spp.* from the Central Balkans. Also, Silva et al.^4^ studied the correlation of the TP of different factions of *H. perforatum* L. extract with the antioxidant activity. They reported that fractions composed of flavonol glycosides contributed the most to the antioxidant activity of the extract.

In this section, we use statistical and chemometric methods to reveal the relations between TP and TP and the DPPH, ORAC and FRAP assays results.

### Linear regression for TP/TF and antioxidants assays

Linear models of TP and TF against FRAP, ORAC and DPPH were created, separately for the extracts made of air-dried and lyophilized materials. For the extracts prepared from air-dried material, the total variances in TP and TF contents are best explained by FRAP assay (67.2%, adjusted-R 2 = 0.741, p < 10^–12^ and 77.3%, adjusted-R2 = 0.790, p < 10^–14^ correspondently).

Similarly, in extracts prepared from lyophilized material the 76% of the total variance in TP is explained by FRAP (adjusted-R 2 = 0.784, p < 10^–15^), whereas DPPH explains 24.3% of total variance in TF (adjusted-R2 = 0.790, p < 10^–14^).

### Principal component analysis

**Figure 2:**
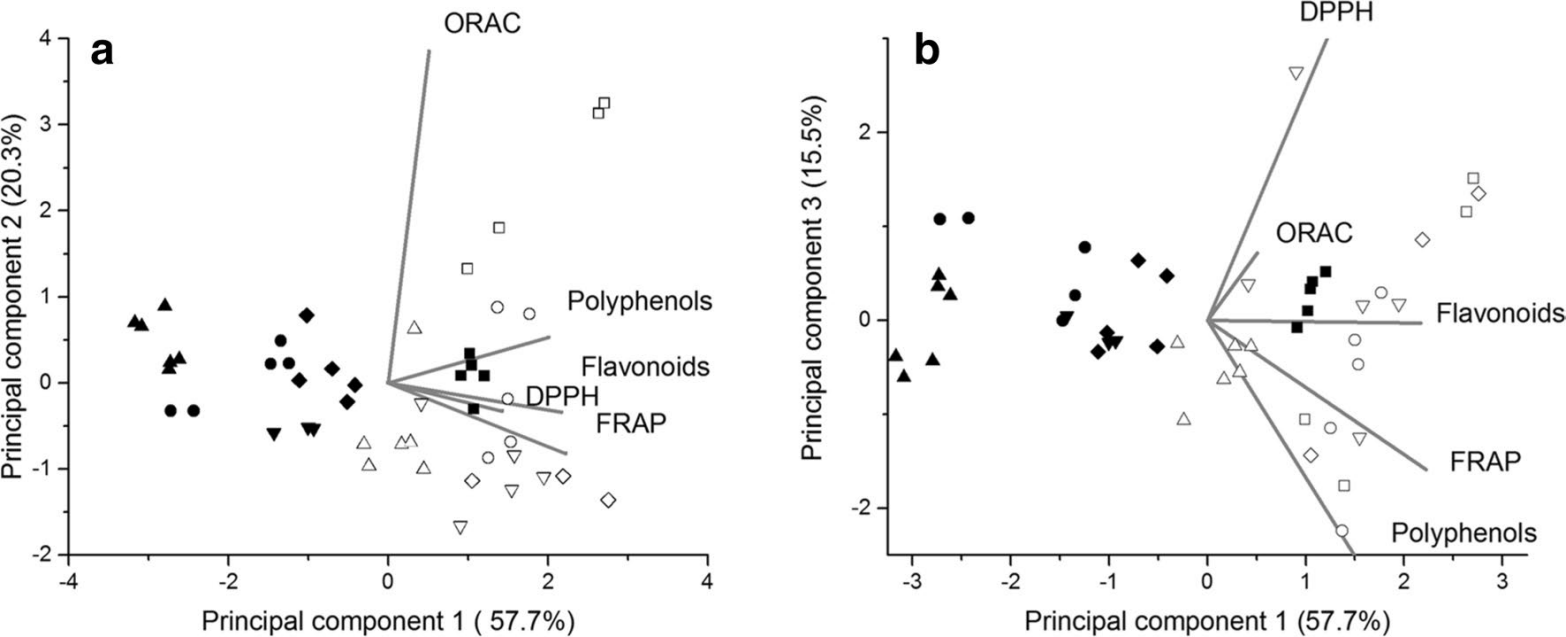
Score plot of principal component 1 vs. principal component 2 **(a)** and of principal component 1 vs principal component 3 **(b)** for the ethanol and ethanol–water extracts of *H. perforatum* L. prepared from dried flowers. Open symbols—ethanol–water extracts, filled symbols—ethanol extracts. Time of collection: 26.06—squares, 20.07—circles, 08.08—triangles, 18.08—diamonds and 28.08—upper triangles.

The first 3 principal components explain more than 90% of sample variation. PC1 is dominated by TP, TF content and FRAP. Variables that contribute most to the separation of the extract along PC2 and PC3 are ORAC and DPPH, respectively (Table 1). In the space of the first 3 principal components, the PCA shows the correlation between TP and TF content and FRAP assay results (Fig. 2). In that case, to describe the properties of ethanol and ethanol–water extracts of *H. perforatum* L. as an effect of harvest time only 3 types of experiments are required i.e. ORAC, DPPH and one of TF, TP or FRAP.

**Table 1:** Loadings of the variables in the space of the first 3 principal components (PC).

Variable	PC1 (57.7%)	PC 2 (20.3%)	PC 3(15.5%)
DPPH	0.36	− 0.08	**0.88**
Polyphenols	**0.50**	0.13	− 0.42
ORAC	0.12	**0.96**	0.09
FRAP	**0.56**	− 0.20	− 0.19
Flavonoids	**0.54**	− **0.08**	− 0.01

Interpretable loadings are given in bold.

The ethanol extracts are distributed along PC1, i.e. the collection date influenced mostly the TF and TP contents and FRAP. On the other hand, the ethanol–water extracts are separated along PC2 and PC3, indicating that, in this case, the collection date influenced mostly DPPH and ORAC values. Such results suggest that compounds other than phenolics, but with a strong radical scavenging activity, are present in ethanol–water extracts.

As described above, both polyphenol and flavonoid content, as well as antioxidant capacity, was higher for extracts prepared from the air-dried plant material than for the ones prepared from the lyophilized one, despite a much lower content of dry matter. This could be due to a less damage to cell walls and tissue structure during the lyophilization compared to other drying methods^42^, which could decrease the penetration of solvent into the plant cells and, in consequence, the extraction of polyphenols and other antioxidants. A lower amount of extracted polyphenols in lyophilized samples, compared to other drying methods, was also observed for herbs from the *Lamiaceae* family^43^. Therefore, even if polyphenols are better preserved during lyophilization than during air-drying, they could be less available for extraction due to better preserved cell walls.

### Erythrocytes studies

Before investigating the antioxidative properties of *H. perforatum* L. extract, we have tested their cytolytic activity against erythrocytes (Fig. S1). The hemolysis values were similar to the control level for all samples at all concentrations, indicating that this model can be used to study the antioxidative activity of our extracts. This is in agreement with the previous results^44^ where it was shown that polyphenol compounds from *H. perforatum* extract alter erythrocyte membrane but do not destroy it even at a high concentrations.

**Figure 3:**
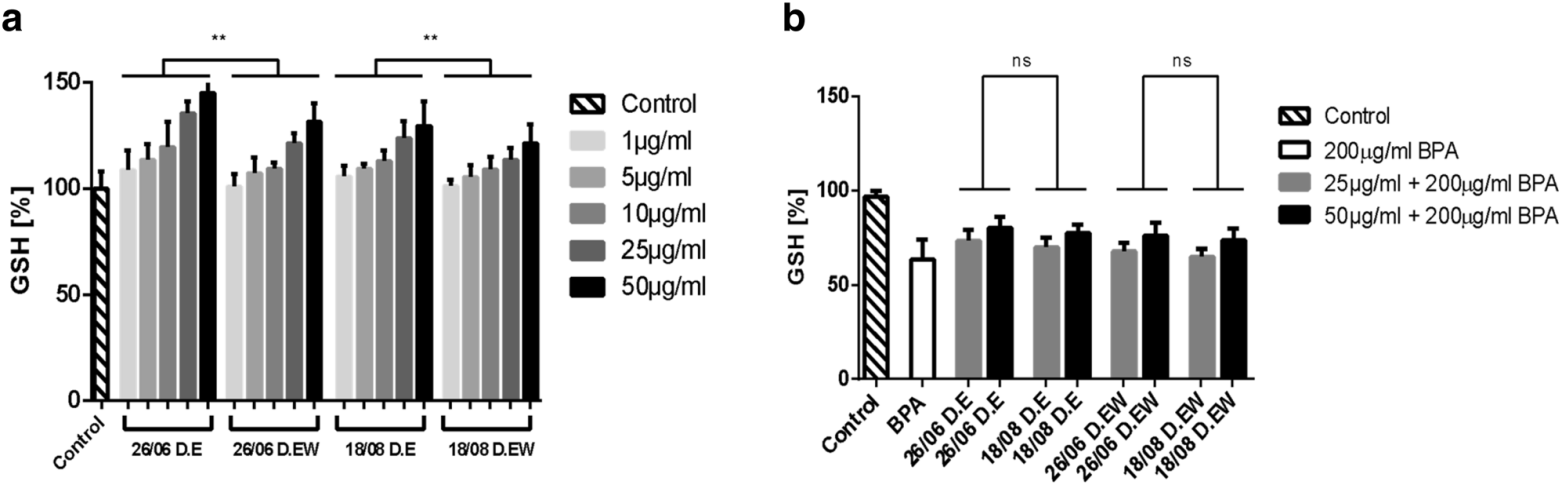
Effect of ethanol (D.E) and ethanol–water (D.EW) extracts of *H. perforatum* L. prepared from air-dried flowers on: **(a)** GSH level in human erythrocytes, **(b)** GSH depletion in human erythrocytes induced by 200 μg/mL BPA.

Ethanol and ethanol–water extracts prepared from the dried material collected on 26.06 and 18.08 (corresponding to the highest polyphenol or flavonoid content) were tested for their effect on the glutathione (GSH) levels in human erythrocytes. Both extracts increased the level of GSH in a concentration-dependent manner (Fig. 3a, see Supporting material for statistical details).

Ethanol extracts increased the GSH level more than ethanol–water ones (S6–S8), in agreement with previous studies^45^ where ethanol extracts of 6 studied plants provided better protection of erythrocyte GSH levels than corresponding aqueous extracts. At a concentration of 50 µg/ml, the strongest effect on the GSH level was observed for the ethanol extract from flowers collected on 26.06. According to Oszmianski et. al.^44^, the polyphenols from *H. perforatum* extract protect erythrocytes by incorporating into the lipid phase of the erythrocyte membrane and increasing its osmotic resistance.

In the presence of 200 µg/ml BPA, the selected extracts were able to partly recover the GSH level (Fig. 3b). Increasing the concentration of ethanol and ethanol–water extracts improved the GSH level recovery (Table S6). No significant effect of collection date (Table S7) and no significant effect of solvent (Table S8) were observed.

Our results show that *H. perforatum* L. extracts could potentially protect erythrocytes from the oxidative damage induced by BPA. However, this protective effect is less pronounced than the one reported for the tannins from the leaves of *Rhus typhina* L^28^.

#### NMR

**Figure 4:**
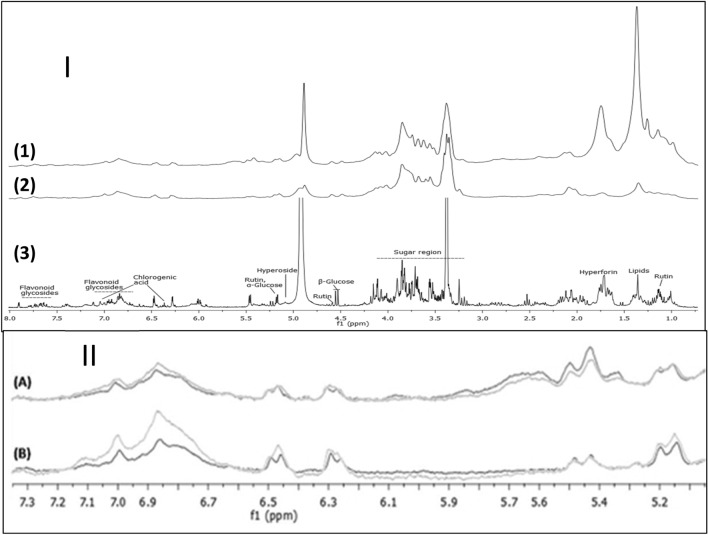
1H NMR spectra of *H. perforatum* L. extracts: (I) benchtop NMR (60 MHz) (1—ethanol extract, 2—ethanol–water extract) versus high field NMR (300 MHz) (3) spectra; (II) ethanol **(A)** and ethanol–water **(B)** extracts prepared from air-dried flowers of *H. perforatum* L. for different harvest time.

We have used two NMR instruments to analyze samples from ethanol and ethanol–water based extraction for two collection dates, June 26 and August 18 (Fig. 4). The first instrument was a conventional spectrometer with 300 MHz frequency, while the second one was a benchtop instrument with 60 MHz frequency. Apart from the analysis of the spectra, we aimed to investigate the extent to which the low-resolution benchtop instrument can be used to analyze samples after prior identification of signals on the higher-resolution 300 MHz spectra.

The assignments of NMR signals of the main compounds were based on the data published by Rasmussen et al.^24^ and Bilia et al.^22^ The 300 MHz spectra with assigned signals are illustrated in Fig. 4. I (3). The analysis showed that the studied extracts contain hyperforin, sugars, lipids, flavonoid glycosides (including such constituents of *H. perforatum* L. extracts such as hyperoside and rutin), and chlorogenic acid.

The spectra recorded with the benchtop NMR (60 MHz) have a lower resolution and consist of highly overlapping signals (Fig. 4 I (1, 2) and Fig. 4 II**A,B**). Nevertheless, 60 MHz NMR shows the differences in the ethanol and ethanol–water extracts, as well as the differences in the extract composition for different collection dates (Fig. 4 II**A,B**).

To compare the samples quantitatively, we have normalized the spectra by their total area, integrated the assigned signals, and computed the ratios of their areas (summarized in Table S9, high field spectra only). Compared to the ethanol extracts, the ethanol–water ones contained less lipids, hyperforin, and hyperoside than the ethanol–water ones, but more sugars, flavonol glycosides and chlorogenic acid. The most pronounced changes from June 26 to August 18 were a decrease in the amount of chlorogenic acid, hyperoside, hyperforin and flavonol glycosides, as well as an increase in the sugar content, in particular the alpha- and beta-glucose. The ethanol extracts also showed a slight increase in the lipid content.

On the other hand, the ethanol–water extracts showed a decrease in the signal in the lipid region. However, as the latter extracts contained a much smaller amount of lipids (between three to five times less than the ethanol ones, see Table S9), we can conclude that the lipid content has increased from June 26 to August 18.

The increase in lipid content is in agreement with the results reported by Amira et al.^46^, where the flowers harvested at the end of August presented higher values of lipids (25.48%) than harvested at the end of June (18.56%). Similar results were reported for the leaf extracts of *Ilex paraguariensis*, where a higher content of fatty acids was found in autumn and winter compared to spring and summer. This could be associated with biotic and abiotic stresses or plant hormones, especially jasmonic acid and its derivatives^47^.

**Figure 5:**
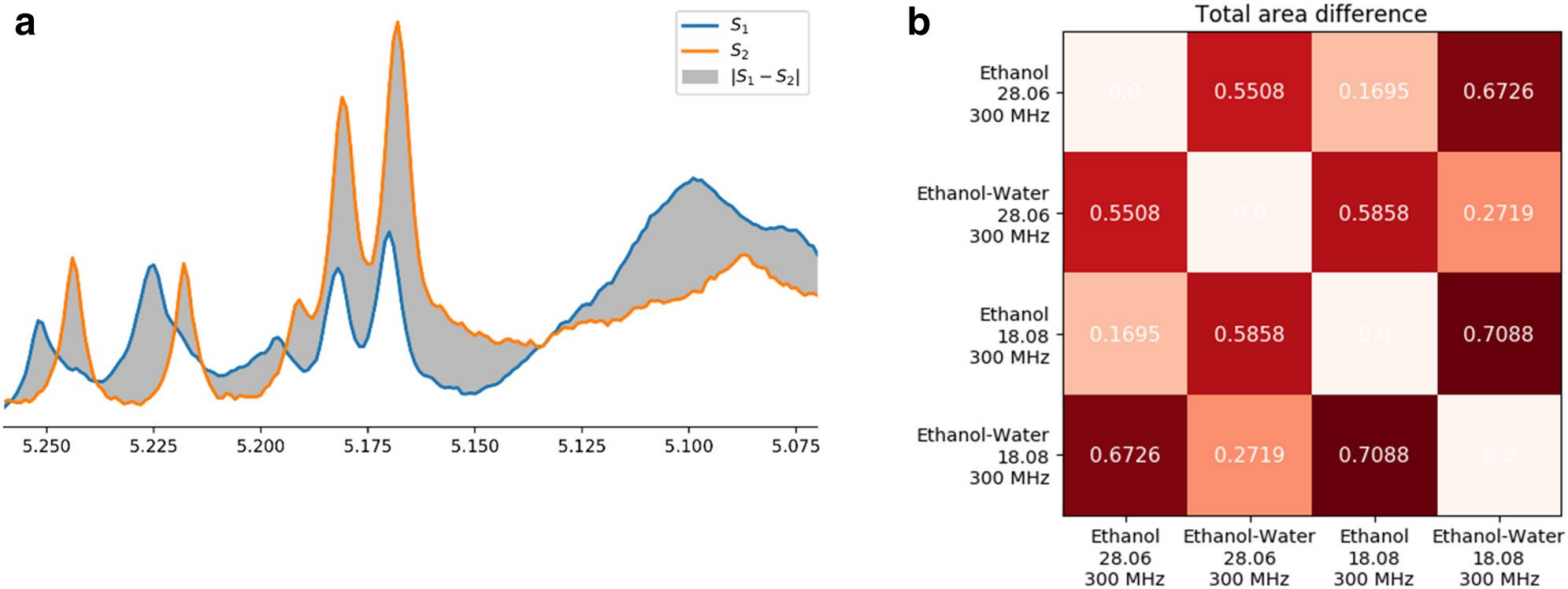
**(a)** The illustration of the L^1^ distance. The blue and orange lines represent two overlayed NMR spectra, S_1_ and S_2_, each normalized by its total area. The L^1^ distance, which measures the overall difference in compound quantities between the spectra, is defined as the area of the region contained between both spectra, highlighted in grey. This distance equals 0 for identical spectra, and equals 2 for spectra with no common signals. The latter value follows from the fact that, in such case, the distance is equal to the sum of areas of both normalized spectra. In the illustrated case, the distance is equal 0.40, indicating a moderately large difference in compound quantities. **(b)** Comparison of NMR spectra obtained on the 300 MHz instrument for different solvents and collection dates. The L1 distance (total area difference) measures the overall difference in signal intensities between the compared spectra. The results indicate that the solvent has a two to four times larger impact on the overall sample composition than the collection date.

In general, the choice of solvent had a much more pronounced effect on the sample composition than the collection date. To check the differences between samples, we have calculated their L^1^ distance, defined as the area contained between the spectra (i.e. the integral of the absolute difference of their signals). This distance measures the overall difference in signals of all compounds. The idea behind it is illustrated in Fig. 5a. The differences between samples obtained using different solvents were two- to four times larger than between different collection dates, as shown in Fig. 5b. The ethanol spectra had only a minor difference between collection dates, with the L^1^ distance approximately equal 0.17. On the other hand, the composition of samples was highly dependent on the solvent choice, as the L^1^ distance between the corresponding spectra reached 0.70.

Our final goal was to check if similar results can be obtained on a 60 MHz benchtop instrument. We have focused on signals which could be identified based on the 300 MHz spectra. Note that, due to much broader signals and an increased J-coupling, the identified regions were broader than in the case of 300 MHz spectra and only a few of them did not overlap with other signals. Thus, the integral regions were adapted as shown in Table 2 and Fig. S2. The 60 MHz instrument has correctly detected the increases and decreases of the quantities of analyzed compounds. Moreover, we have detected a statistically significant positive correlation (0.9) between the 300 MHz and 60 MHz results when different solvents and different dates were compared.

**Table 2:** Comparison of signal area ratio changes measured on 300 MHz and 60 MHz instruments. Increasing and decreasing signals highlighted in bold and italics respectively.

			Signal area ratios			
			Ethanol–water to ethanol			
Compound	ppm range		August 18		June 28	
	300 MHz	60 MHz	300 MHz	60 MHz	300 MHz	60 MHz
Rutin and lipids	0.75–1.55	0.7–1.55	*0.231*	*0.289*	*0.356*	*0.324*
Hyperforin	1.6–1.8	1.57–1.92	*0.223*	*0.307*	*0.439*	*0.286*
Beta-glucose	4.51–4.57	4.44–4.66	**1.328**	**1.602**	**1.437**	**1.853**
Chlorogenic acid	6.24–6.31	6.16–6.34	**1.295**	**1.627**	**1.356**	**1.780**
Flavonoid glycosides	6.55–7.26	6.55–7.26	**1.271**	**1.833**	**1.490**	**1.974**
	7.88–7.93	7.85–7.96	**1.177**	**2.302**	**1.002**	**2.091**
Correlation			0.921988		0.905054	
p-value			< 0.005		< 0.005	

Our results suggest that a low-field benchtop instrument can be used to detect changes in the sample composition when signals are identified on a high-field instrument and carefully adjusted for low-field spectra. Further development of statistical methodology, such as confidence intervals for area ratios, is also needed in order to obtain more reliable results with low-field benchtop instruments. If this is achieved, such instruments have the potential to greatly reduce the time and costs of preliminary analyses and screenings of samples.

## Conclusions

Ethanol and ethanol–water extracts of dried and lyophilized flowers of *H. perforatum* L. from Poland showed the dependency of TP and TF contents, as well as of the antioxidant properties, on the collection time. Such dependency could potentially be correlated with the average temperatures during the day. The optimal collection time differed for TP and TF content, being 26.06 and 18.08, respectively. The drying method had a significant effect on the TP and TF content and antioxidant capacity. Generally, the extracts prepared from room temperature air-dried flowers had higher content of bioactive compounds and higher antioxidant capacity than those prepared from lyophilized flowers. Principal Component Analysis showed that TP and TF content is mostly correlated with FRAP values and suggested that non-phenolic compounds also contribute to the DPPH radical scavenging and total antioxidant capacity of the ethanol–water extract. Further studies are needed in order to determine these compounds. The ethanol and ethanol–water extracts of *H. perforatum* L. were able to protect GSH from the depletion in the BPA-induced oxidative stress. The ethanol extracts were slightly more effective than ethanol–water extracts, contrary to ORAC, DPPH and FRAP results. Our study also showed that spectra collected on a low-field benchtop NMR spectrometer can be used to evaluate the composition of the extracts if the assignment of signals is done previously on an representative extract using a high field NMR spectrometer and if the database on chemical shifts is available.

## Material and methods

Plant material (flowering tops) was collected from its natural habitat in 2016 from the end of June till the end of August (26.06, 20.07, 8.08, 18.08, 28.08) in the vicinity of Radom (Mazovia Province), located in the east-central of Poland (GPS 51.317709, 21.259254). The place is 162 m above sea level, and it is naturally sunny place. According to Köppen-Geiger climate classification this climate is classified as Dfb (warm-summer humid continental climate). The plant material was compared with the botanical description key^48^ and the botanical drawing from the atlas of plants (http://www.biolib.de/). The shape of the stem and leaves, the arrangement of the leaves and the inflorescence, the structure / type of flower, the appearance of the fruit was compared. The characteristic feature of the *H.perforatum* L. i.e. translucent dots on flower petals and leaf blades were identified. After identification of the plant, only flowers at the blooming stage were collected. Part of the collected plant material was air-dried in a dark place, at room temperature for 7 days. The second part of the collected plant material was frozen and lyophilized (at − 25 °C for 96 h). The water content of dried plant material was determined by the oven-drying method. The part of dried and lyophilized plant materials were weighted , dried in an oven for 2 h at 105 °C and reweighed. Then 50 ml of solvent (ethanol 96% and ethanol–water (1:1)) was added to 1 g of mechanically ground plant material and sonicated at 30 ºC over 15 min. Then, extracts were filtered and dried in vacuum at 25 ºC (ethanol) or 35 ºC (ethanol–water). For analysis, reconstituted solution (concentration of 2 mg/ml) were used. All samples were prepared in duplicates. The prepared extracts were kept at − 18 ºC until used.

### Total polyphenol (TP) and total flavonoid (TF) content

TP content has been determined using the modified Folin-Ciocalteu method^49^. Results were expressed as mg of gallic acid equivalents per 1 g of total extract weight (mg GAE/g). TF content has been determined by the AlCl3 colorimetric method^50^ and expressed as mg of catechin equivalents per 1 g of total extract weight (mg CAE/g). All experiments were done in triplicate.

### Antioxidant capacity assays: ORAC, DPPH (EPR) and FRAP

All experiments were done in triplicate. The ORAC assay using fluorescein was based on Ou et al.^51^ Results were expressed as µmol of Trolox per 1 g of extract (μmol TE/g). The free radical scavenging activity of the extracts has been measured by DPPH (EPR) assay. An extract solution (10–30 μl) or a blank was mixed with 1.3 mM DPPH solution (200 μl). After 30 min EPR spectra were recorded, using Miniscope MS200 spectrometer (Magnettech GmbH, Germany), with following parameters: microwave power 10 mW, modulation amplitude 0.1 mT, central field 334 mT, sweep time 30 s, sweep range 8 mT. Results were expressed as mg of scavenged DPPH per g of extract (mg DPPH/g). The FRAP assay has been done according to Benzie^52^. The results were expressed as mmol of produced Fe^2+^ per 1 g of extract (mmol Fe^2+^/g). All results are given for the whole extract weight.

#### NMR

The extracts (10 mg) were dissolved in 400 µl of deuterated methanol (CD_3_OD). 1H NMR spectra were recorded at 301 K on a Magritek 60 MHz Ultra Spinsolve instrument (Magritek GmbH, Aachen, Germany) (256 scans, a repetition time of 15 s, an acquisition time of 6.4 s with the suppression of the water peak) or a Varian VNMRS 300 Oxford spectrometer (Agilent Technologies, Santa Clara, USA) operating at 299.61 (1H) MHz (128 scans, a repetition time of 1 s and an acquisition time of 2 s). Each sample was prepared in duplicate. The spectra were analyzed using the MestReNova program (Mestrelab Research S.L.).

### Determination of GSH content

Anticoagulated blood samples were kindly provided by the Regional Blood Center in Bialystok. The study is approved by the ethics committee of the Medical University of Bialystok (R-I-002/77/2015). The erythrocyte isolation was performed as described earlier^28^. 1 ml of 1% suspension of erythrocytes (PBS, pH 7.4) has been incubated with *H. perforatum* L. extract at 37 °C for 60 min. Next, the suspension was incubated for 4 h with 200 µg/ml BPA and then 0.2 ml of 25% trichloroacetic acid was added. For the reference studies of *H. perforatum* L. extract influence on GSH content, the step with incubation with BPA was omitted. After incubation, the samples were centrifuged (400*g*, 10 min). To 0.5 ml of the supernatant, 0.5 ml 0.5 M phosphate buffer (pH 7.8) and 0.05 ml Ellman’s reagent (5 mM) were added. After 30 min the samples were monitored spectrophotometrically at 414 nm^53^.

### Statistical analysis

The statistical significance of the harvest time dependency was evaluated with pairwise T-test and ANOVA. The statistical difference among five groups defined by specific harvest times was evaluated. Tests were performed separately for polyphenols and flavonoids in both studied solvents: ethanol and ethanol water, resulting in four testing assessments. Additionally, the linear regression analysis was used to test if the assays values significantly predicted TP/TF content in all observations. To encode the statistical significance (with respect to p-values) asterisks are used: (****)—p < 0.0001; (***)—p < 0.001; (**)—p < 0.01; (*)—p < 0.05; (ns)—p 0.05 (no statistical evidence).

### Principal component analysis (PCA)

PCA has been performed in Matlab R2017, using “princom” function. Prior to PCA, all data (TP, TF, FRAP, ORAC and DPPH) were normalized using “zscore” function.

## Supplementary Information

Supplementary Information 1.

## Abbreviations

BPA: 2,2-Bis(4-hydroxyphenyl)propane
DPPH: 2,2′-Diphenyl-1-picrylhydrazyl
EPR: Electron paramagnetic resonance
FRAP: Ferric reducing antioxidant power
GSH: Glutathione
NMR: Nuclear magnetic resonance
ORAC: Oxygen radical absorbance activity
PBS: Phosphate-buffered saline
PC: Principal component
PCA: Principal component analysis
TF: Total flavonoids
TP: Total polyphenols
TE: Trolox
