# Supplementary material for: Harvest time affects antioxidant capacity, total polyphenol and flavonoid content of Polish St John’s wort’s (Hypericum perforatum L.) flowers
Source: Sci Rep. 2021 Feb 17;11:3989. doi: 10.1038/s41598-021-83409-4 (PMC7889936; doi:10.1038/s41598-021-83409-4)
Supplement: Supplementary file 1 — Supplementary Information 1. [file 41598_2021_83409_MOESM1_ESM.docx]

**Harvest time affects antioxidant capacity, total polyphenol and flavonoid content of Polish St John’s wort’s (*Hypericum perforatum* L.) flowers.**

*Katerina Makarova^1*^, Joanna J. Sajkowska-Kozielewicz^1^, Katarzyna Zawada^1^, Ewa Olchowik-Grabarek^2^, Michał Aleksander Ciach^3,4^ , Krzysztof Gogolewski^3^, Natalia Dobros^1^, Paulina Ciechowicz^1^, Helene Freichels^5^, Anna Gambin^3^*

**Supplementary Materials**

The following document contains an extended description of all the statistical analysis that was done for the research by Makarova et al. The document describes all the work that was performed on the set of 120 samples described by 5 measured continuous variables (TP, TF, FRAP, DPPH, ORAC) along with 3 categorical variables (harvest time, solvent type, drying method) described in the main article.

**Basic statistics**

To reduce the influence of outliers on the basic statistics, below we present medians instead of means provided along with the standard deviation (sd) and min/max observed values. In the brackets there are p-values that correspond to the two-sample T-test that verified the statistical significance of the difference in means for ethanol-water vs ethanol extracts.

**Table S1. T**h**e** basic statistics of TP and TF for *H. perforatum* L. flowers extracts

|  |  | TP content [mg GAE/g] | | | TF content [mg CAE/g] | | |
| --- | --- | --- | --- | --- | --- | --- | --- |
|  |  | Median±SD | Min | Max | Median±SD | Min | Max |
| Air-dried samples | Ethanol-water | 371 ±49 | 317.6 | 402.2 | 160 ± 7 | 138.4 | 175.3 |
|  | Ethanol | 245 ± 26 | 199.6 | 298.2 | 122 ± 4 | 100.4 | 167.8 |
|  | p | < 10^-11^ | | | < 10^-6^ | | |
| Lyophilized | Ethanol-water | 238± 26 | 214.2 | 302.4 | 107 ± 16 | 96.0 | 151.0 |
|  | Ethanol | 152 ± 13 | 135.7 | 156.1 | 80 ± 7 | 69.6 | 85.0 |
|  | p | < 10^-14^ | | | < 10^-10^ | | |

**Table S2.** P-values for pairwised T-test for TP and TF along with 3 categorical variables (harvest time, solvent type, drying method), p <0.05 are in bold

|  |  | **DRY SAMPLES** | | | | | **LYOPHILIZED SAMPLES** | | | | |  |
| --- | --- | --- | --- | --- | --- | --- | --- | --- | --- | --- | --- | --- |
| **FLAVANOIDS** | **ETHANOL** |  | **26.06** | **20.07** | **8.08** | **18.08** |  | **26.06** | **20.07** | **8.08** | **18.08** | |
|  |  | **20.07** | **3.90E-15** | - | - | - | **20.07** | **0.0087** | - | - | - | |
|  |  | **8.08** | **2.00E-16** | **1.90E-08** | - | - | **8.08** | **0.0137** | 0.8460 | - | - | |
|  |  | **18.08** | **1.80E-12** | **0.0005** | **6.10E-12** | **-** | **18.08** | **0.0010** | 0.3972 | 0.3003 | - | |
|  |  | **28.08** | **3.90E-14** | 0.1206 | **5.90E-10** | **0.0248** | **28.08** | 0.5853 | **0.0304** | **0.0460** | **0.0041** | |
|  | **ETHANOL-H2O** |  | **26.06** | **20.07** | **8.08** | **18.08** |  | **26.06** | **20.07** | **8.08** | **18.08** | |
|  |  | **20.07** | 0.60315 | - | - | - | **20.07** | **5.30E-09** | - | - | - | |
|  |  | **8.08** | **0.00013** | **3.20E-05** | **-** | - | **8.08** | 0.99209 | **5.10E-09** | - | - | |
|  |  | **18.08** | **0.00364** | **0.01279** | **4.30E-08** | - | **18.08** | 0.10515 | **2.50E-07** | 0.10320 | - | |
|  |  | **28.08** | 0.74580 | 0.40103 | **0.00029** | **0.00161** | **28.08** | **0.02847** | **4.60E-11** | **0.02910** | **0.00049** | |
| **POLYPHENOLS** | **ETHANOL** |  | **26.06** | **20.07** | **8.08** | **18.08** |  | **26.06** | **20.07** | **8.08** | **18.08** | |
|  |  | **20.07** | **5.50E-06** | - | - | - | **20.07** | 0.4960 | - | - | - | |
|  |  | **8.08** | **1.00E-05** | 0.8028 | - | - | **8.08** | 0.7780 | 0.6880 | - | - | |
|  |  | **18.08** | **0.0370** | **0.0016** | **0.0030** | - | **18.08** | 0.7480 | 0.7180 | 0.9680 | - | |
|  |  | **28.08** | **0.0402** | **0.0014** | **0.0027** | 0.9693 | **28.08** | **0.0200** | **0.0860** | **0.0380** | **0.0410** | |
|  | **ETHANOL-H2O** |  | **26.06** | **20.07** | **8.08** | **18.08** |  | **26.06** | **20.07** | **8.08** | **18.08** | |
|  |  | **20.07** | 0.4398 | - | - | - | **20.07** | **9.10E-06** | - | - | - | |
|  |  | **8.08** | **0.0158** | **8.31E-02** | - | - | **8.08** | 0.58468 | **2.20E-06** | - | - | |
|  |  | **18.08** | 0.3006 | 0.7878 | 0.1378 | - | **18.08** | **0.00694** | **0.01517** | **0.00179** | - | |
|  |  | **28.08** | **0.0043** | **0.0267** | 0.5871 | **0.0476** | **28.08** | 0.16826 | **0.00035** | **0.05969** | 0.14036 | |

*Table S3 Average air temperatures in June-August 2016*

| **Period** | **16.06-26.06** | **10.07-20.07** | **28.07-08.08** | **08.08-18.08** | **18.08-28.08** |
| --- | --- | --- | --- | --- | --- |
| **Temperature, average high, C** | **29** | 23 | **26** | 22 | **26** |
| **Temperature, average low, C** | 15 | 15 | 15 | 11 | 11 |

**Table S4.** Th**e** basic statistics of DPPH, ORAC and FRAP assays for *H. perforatum* L. flowers extracts

|  |  | DPPH [mg DPPH/g] | | | ORAC [µmol TE/g] | | | | | FRAP [mmol Fe^2+^/g] | | |
| --- | --- | --- | --- | --- | --- | --- | --- | --- | --- | --- | --- | --- |
|  |  | Median±SD | Min | Max | | Median±SD | Min | Max | Median±SD | | Min | Max |
| Air-dried samples | Ethanol-water | 1672±178 | 1647 | 2306 | | 5214±2167 | 5141 | 33111 | 2.54±0.34 | | 2.27 | 2.87 |
|  | Ethanol | 1378±149 | 1290 | 1920 | | 9701±3430 | 6325 | 14713 | 1.39±0.30 | | 1.26 | 2.42 |
|  | p | NS | | | | NS | | | < 0.05 | | | |
| Lyophilized | Ethanol-water | 1287±123 | 947 | 1876 | | 3313±514 | 3282 | 4893 | 0.31±0.01 | | 0.30 | 0.31 |
|  | Ethanol | 653±51 | 628 | 812 | | 775±293 | 863 | 1704 | 0.63±0.02 | | 0.61 | 0.65 |
|  | p | < 0.05 | | | | < 0.05 | | | < 0.05 | | | |

**Table S5**. P-values for pairwised T-test for FRAP, DPPH and ORAC along with 3 categorical variables (harvest time, solvent type, drying method), p <0.05 are in bold

|  |  | **DRY SAMPLES** | | | | | **LYOPHILIZED SAMPLES** | | | | |  |
| --- | --- | --- | --- | --- | --- | --- | --- | --- | --- | --- | --- | --- |
| **DPPH** | **ETHANOL** | date | **26.06** | **20.07** | **8.08** | **18.08** | date | **26.06** | **20.07** | **8.08** | **18.08** |  |
|  |  | **20.07** | 0.0568 | - | - | - | **20.07** | 0.53704 | - | - | - |  |
|  |  | **8.08** | **9.20E-06** | **0.0012** | - | - | **8.08** | **0.00537** | **0.00016** | - | - |  |
|  |  | **18.08** | **0.0178** | 0.6431 | **0.0024** | - | **18.08** | 0.08959 | **0.00752** | 0.12212 | - |  |
|  |  | **28.08** | **0.0147** | 0.3693 | **0.033** | 0.5912 | **28.08** | 0.19999 | 0.40439 | **2.00E-05** | **0.001** |  |
|  | **ETHANOL-H2O** | date | **26.06** | **20.07** | **8.08** | **18.08** | date | **26.06** | **20.07** | **8.08** | **18.08** |  |
|  |  | **20.07** | 0.267 | - | - | - | **20.07** | **2.90E-08** | - | - | - |  |
|  |  | **8.08** | 0.193 | 0.88 | - | - | **8.08** | **0.0034** | **0.00039** | - | - |  |
|  |  | **18.08** | 0.744 | 0.201 | 0.151 | - | **18.08** | 0.33734 | **9.00E-09** | **0.00054** | - |  |
|  |  | **28.08** | 0.468 | 0.067 | **0.041** | 0.773 | **28.08** | **0.00015** | **9.60E-12** | **2.20E-07** | **0.00269** |  |
| **ORAC** | **ETHANOL** |  | **26.06** | **20.07** | **8.08** | **18.08** |  | **26.06** | **20.07** | **8.08** | **18.08** |  |
|  |  | **20.07** | **0.00543** | - | - | - | **20.07** | 0.8918 | - | - | - |  |
|  |  | **8.08** | **1.66E-01** | 0.09748 | - | - | **8.08** | 0.1627 | 0.2759 | - | - |  |
|  |  | **18.08** | 0.24529 | 0.07981 | 0.85389 | - | **18.08** | **0.0054** | **0.018** | 0.1029 | - |  |
|  |  | **28.08** | **1.80E-05** | **0.01583** | **0.00032** | **0.00032** | **28.08** | 0.4317 | 0.4129 | **0.0435** | **0.0014** |  |
|  | **ETHANOL-H2O** |  | **26.06** | **20.07** | **8.08** | **18.08** |  | **26.06** | **20.07** | **8.08** | **18.08** |  |
|  |  | **20.07** | **1.30E-05** | - | - | - | **20.07** | 0.87157 | - | - | - |  |
|  |  | **8.08** | **4.00E-07** | 0.103 | - | - | **8.08** | **0.01472** | **0.02055** | - | - |  |
|  |  | **18.08** | **3.30E-07** | 0.087 | 0.928 | - | **18.08** | **0.00281** | **0.00409** | 0.59459 | - |  |
|  |  | **28.08** | **3.30E-07** | 0.057 | 0.7 | 0.765 | **28.08** | **0.0003** | **0.00044** | 0.2005 | 0.42633 |  |
| **FRAP** | **ETHANOL** |  | **26.06** | **20.07** | **8.08** | **18.08** |  | **26.06** | **20.07** | **8.08** | **18.08** |  |
|  |  | **20.07** | **1.30E-09** | - | - | - | **20.07** | 0.47888 | - | - | - |  |
|  |  | **8.08** | **9.10E-11** | 0.19371 | - | - | **8.08** | 0.29118 | 0.19371 | - | - |  |
|  |  | **18.08** | **0.00091** | **9.20E-06** | **3.30E-07** | - | **18.08** | 0.22 | **9.20E-06** | **3.30E-07** | - |  |
|  |  | **28.08** | **0.00033** | **2.60E-05** | **9.70E-07** | 0.69317 | **28.08** | **0.01238** | **2.60E-05** | **9.70E-07** | 0.69317 |  |
|  | **ETHANOL-H2O** |  | **26.06** | **20.07** | **8.08** | **18.08** |  | **26.06** | **20.07** | **8.08** | **18.08** | |
|  |  | **20.07** | **0.03894** | - | - | - | **20.07** | **0.03894** | - | - | - | |
|  |  | **8.08** | 0.12382 | **0.00089** | - | - | **8.08** | 0.12382 | 0.08439 | - | - | |
|  |  | **18.08** | **0.00016** | **0.03256** | **2.60E-06** | - | **18.08** | **0.00016** | 0.59454 | **0.02779** | - | |
|  |  | **28.08** | 0.30101 | 0.27203 | **0.0138** | **0.00235** | **28.08** | 0.30101 | **0.00219** | 0.11832 | **0.00056** | |

**Erythrocytes studies**

**

**

**Fig. S1** Effect of ethanol (D.E) and ethanol-water (D.EW) extracts of *H. perforatum* L. prepared from air-dried flowers on percentage of the hemolysis of human erythrocytes.

**GSH levels in human erythrocytes**

Using two-sample paired T-test the difference between two solvent types (ethanol vs ethanol-water) across the concentration value for both time-points were statistically confirmed.

Specifically, we perform the test for averaged values among all studied concentrations. Our hypothesis is that Ethanol solvent brings greater average of GSH percent than Ethanol-water

- For 26.06 a mean of differences (between ethanol and ethanol-water) is equal to 9.999967 for 95% confidence interval of values greater than 6.974844, which gives a p-value=0.0011
- For 18.08 a mean of differences is equal to 6.139683 for 95% confidence interval of values greater than 3.312151, which gives a p-value=0.0049

**GSH levels in human erythrocytes in the presence of 200 µg/ml BPA**

The two-sample (unpaired) T-test was performed to check if the mean is greater in a statistically significant manner between two samples. Tested scenarios and p-values are put in table S7-S9:

We question the level of GSH. The hypothesis is that the 50 µg/ml extracts gave significantly higher GSH values than 25 µg/ml ones.

**Table S6. Two-sided unpaired t-test p-values** for GSH depletion in the presence of BPA and *H. perforatum* L. flowers extracts **calculated for the same solvent and same date, only measurements with different values of C [µg/ml] are compared**

| **SOLVENT** | **DATE** | **PVALUE** |
| --- | --- | --- |
| Ethanol | 26.06 | 0.06723083 |
| Ethanol | 18.08 | 0.01686112 |
| Ethanol-water | 26.06 | 0.03356959 |
| Ethanol-water | 18.08 | 0.01621146 |

**Table S7 Two-sided unpaired t-test p-values**  for GSH depletion in the presence of BPA and *H. perforatum* L. flowers extracts **calculated for the same solvent and same C [µg/ml], only measurements from different dates are compared.**

| **SOLVENT** | **C [µg/ml]** | **PVALUE** |
| --- | --- | --- |
| Ethanol | 25 | 0.3367115 |
| Ethanol | 50 | 0.4011165 |
| Ethanol-water | 25 | 0.2770239 |
| Ethanol-water | 50 | 0.5330021 |

**Table S8. Two-sided unpaired t-test p-values**  for GSH depletion in the presence of BPA and *H. perforatum* L. flowers extracts **calculated for same date and same C [µg/ml], only measurements from different solvents are compared**

| **DATE** | **C [µg/ml]** | **PVALUE** |
| --- | --- | --- |
| 26.06 | 25 | 0.1198361 |
| 18.08 | 50 | 0.3039367 |
| 26.06 | 25 | 0.0942443 |
| 18.08 | 50 | 0.2437111 |

**NMR analysis**

| 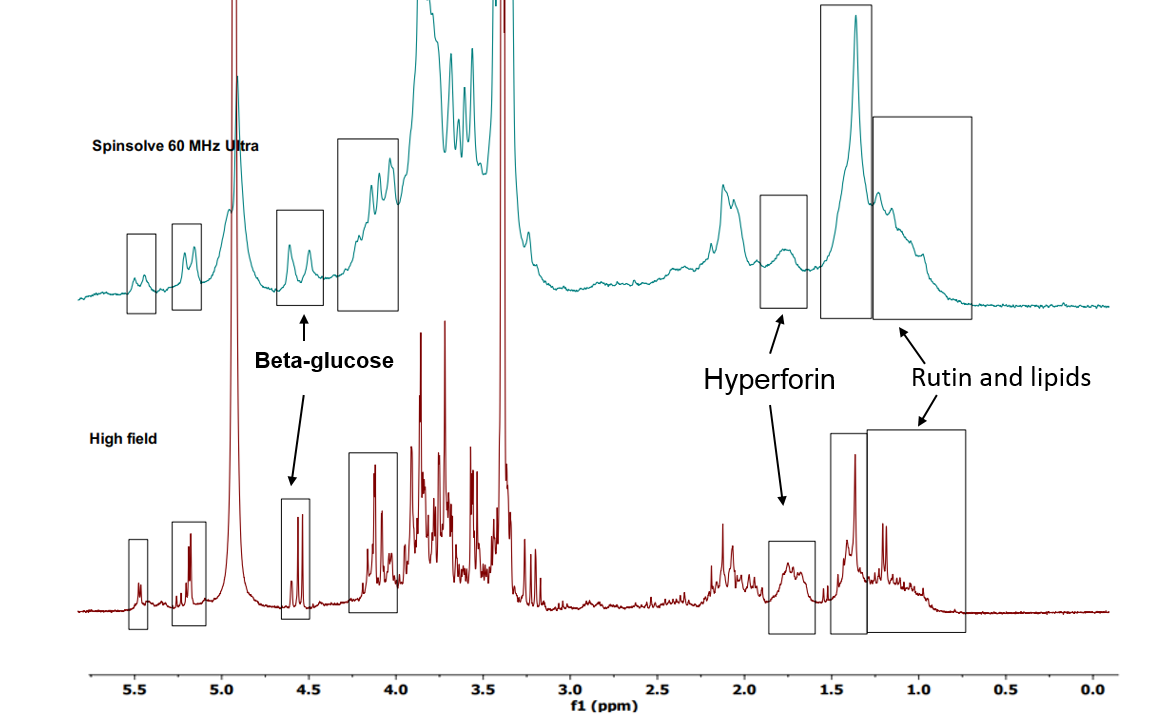 |
| --- |
| 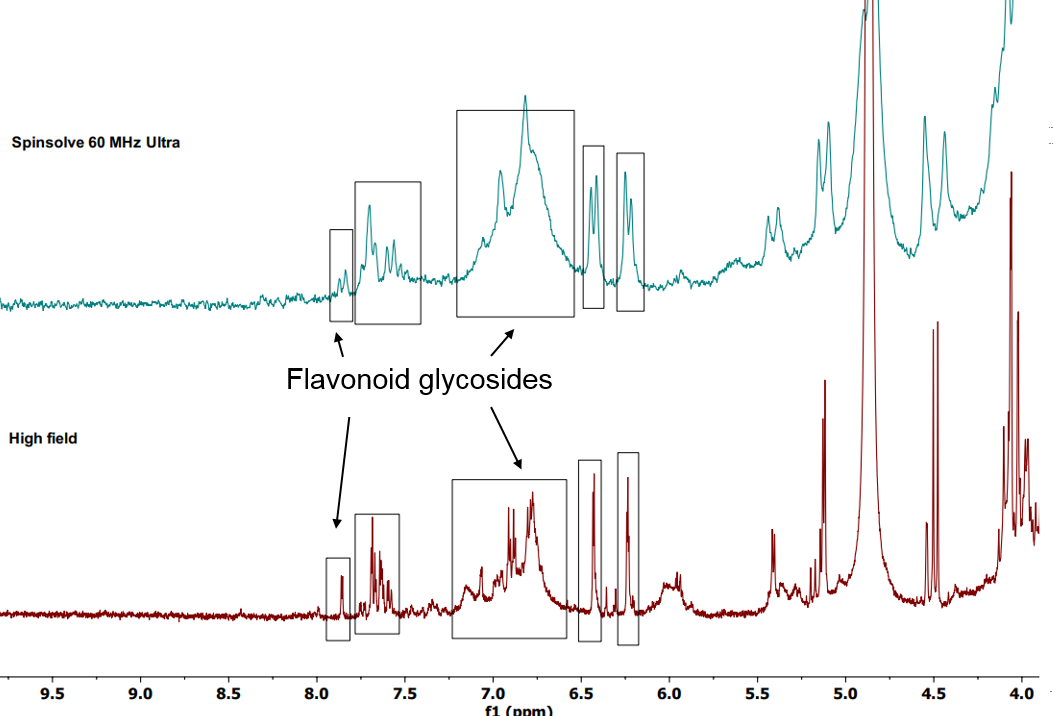 |

**Fig. S2. Regions selected for the area ratio analysis of low field and high filed NMR spectra**

***Table S9*** *Signal fold changes for identified components computed from 300 MHz spectra. Increasing and decreasing signals highlighted in green and blue respectively.*

|  | | **Signal area ratio** | | | |
| --- | --- | --- | --- | --- | --- |
|  |  | **August 18 to June 28** | | **Ethanol-Water to Ethanol** | |
| **Compound** | **ppm range** | **Ethanol-Water** | **Ethanol** | **August 18** | **June 28** |
| Rutin | 0.85-1.06 | 0.607 | 0.967 | 0.256 | 0.407 |
| Lipids | 1.07-1.3 | 0.678 | 1.224 | 0.187 | 0.338 |
| Hyperforin | 1.6-1.8 | 0.501 | 0.985 | 0.223 | 0.439 |
| Beta-glucose | 4.51-4.57 | 1.257 | 1.361 | 1.328 | 1.437 |
| Hyperoside | 5.04-5.14 | 0.654 | 0.993 | 0.38 | 0.577 |
| Alpha-glucose | 5.15-5.19 | 1.002 | 1.208 | 1.062 | 1.281 |
| Chlorogenic acid | 6.24-6.31 | 0.747 | 0.782 | 1.295 | 1.356 |
| Flavonoid glycosides | 6.55-7.26 | 0.66 | 0.773 | 1.271 | 1.49 |
|  | 7.88-7.93 | 1.003 | 0.854 | 1.177 | 1.002 |
